# Associations between white matter micro- and macro-structure and attention in 6-7-year-old children with low to moderate prenatal alcohol exposure

**DOI:** 10.1007/s11682-026-01110-4

**Published:** 2026-02-25

**Authors:** Philippa Pyman, Claire E. Kelly, Thijs Dhollander, Garance Delagneau, Elizabeth J. Elliott, Anthony J. Penington, Stephen Hearps, Simonne E. Collins, Sharon Lewis, Xiaoyun Liang, Alicia Spittle, Renee Testa, Jane Halliday, Evelyne Muggli, Deanne K. Thompson, Peter J. Anderson

**Affiliations:** 1 Turner Institute for Brain and Mental Health, Monash University, Melbourne, Australia; 2 Murdoch Children’s Research Institute, Melbourne, Australia; 3 Child and Adolescent Health, Faculty of Medicine and Health, University of Sydney, Sydney, Australia; 4 The Sydney Children’s Hospitals Network, Westmead, Sydney, Australia; 5 Royal Children’s Hospital, Melbourne, Australia; 6 Department of Paediatrics, The University of Melbourne, Melbourne, Australia; 7 Department of Physiotherapy, The University of Melbourne, Melbourne, Australia; 8 Department of Pediatrics, School of Medicine, University of California, Irvine, Orange, USA; 9 Children’s Hospital of Orange County, 1201 W. La Veta, Orange, CA 92868 USA

**Keywords:** Prenatal alcohol exposure, Attention, Brain microstructure, Diffusion-weighted MRI, Fixel based analysis

## Abstract

Heavy prenatal alcohol exposure is associated with changes to the brain’s white matter, which may underpin attention problems observed in this population. Less is known about the effect of low to moderate exposure. This study investigated white matter correlates of attention in children with low to moderate levels of prenatal alcohol exposure and controls whose mothers abstained from alcohol. We also examined whether associations differed between alcohol exposure in trimester one only or throughout pregnancy. A total of 129 children aged 6-7-years from the prospective Asking Questions about Alcohol in pregnancy (AQUA) cohort (trimester one alcohol exposure *n* = 47; exposure throughout pregnancy *n* = 45; controls *n* = 37) participated in a brain magnetic resonance imaging study. Fixel-based analysis was performed on diffusion weighted images to investigate associations between whole brain white matter micro- and macro-structure and attention (focus, shift, sustain, hyperactivity and inattention). In controls, better focused attention and less hyperactivity were associated with greater macrostructural cross-sectional area of the corticospinal tract and cerebellar white matter, respectively. Greater microstructural fiber density in the splenium of the corpus callosum was associated with more inattention in the children with trimester one alcohol exposure, which persisted when adjusted for any binge drinking episodes prior to pregnancy recognition. Subtle differences in the association between white matter and attention were found between controls and prenatal exposure groups, where associations were stronger in controls. While further exploration is needed, preliminary findings in 6-7-year-old children suggest that low to moderate prenatal alcohol exposure affects white matter fibers that subserve attention skills.

**Supplementary Information:**

The online version contains supplementary material available at 10.1007/s11682-026-01110-4.

## Introduction

Despite a high prevalence of low-moderate alcohol intake during pregnancy, the long-term consequences of prenatal alcohol exposure (PAE) remain poorly understood (Muggli et al., 2016; Young et al., 2022). Risks associated with PAE are predominantly informed by children who have Fetal Alcohol Spectrum Disorder (FASD). FASD encompasses cognitive, behavioral, brain and growth deficits that are generally associated with heavy and chronic PAE (Jacobson et al., 2021; Kodituwakku et al., 2011). Attention problems are a core feature and a key diagnostic criterion for FASD diagnosis (Bower & Elliott, 2016). It has been hypothesized that low to moderate PAE have similar, albeit more subtle, effects on neurodevelopment (Wozniak et al., 2019). Findings are variable and few studies have found an association between low to moderate PAE and developmental deficits (Flak et al., 2014; Muggli et al., 2024; Ogunjimi et al., 2025).

Disruption to the brain’s white matter underpins the adverse neurodevelopmental outcomes observed in FASD (Ghazi Sherbaf et al., 2019). A review of diffusion tensor imaging findings identified diffuse white matter changes in FASD, with the most affected regions being the corpus callosum, inferior and superior longitudinal fasciculi, cerebellar peduncles and cingulum (Ghazi Sherbaf et al., 2019). Radiological findings on clinical MRI in FASD patients are inconsistent and dependent on timing dose and duration of PAE (Treit et al., 2020). A dose-response relationship has been identified between PAE and abnormalities of white matter microstructure in the corpus callosum, inviting hypotheses that low levels of PAE have adverse effects (Fan et al., 2016; Fryer et al., 2009; Li et al., 2009). One study found an association between moderate and occasional binge PAE and altered white matter microstructure in the superior longitudinal fasciculus and inferior cerebellar peduncles in infants with PAE compared to unexposed controls (Donald et al., 2015). Given that many of the regions associated with PAE are important for attention (Lebel et al., 2010), findings may have broad clinical implications for cognitive functioning.

Different neural networks are important for focused, shifting and sustained attention (Ware et al., 2021). For example, altered microstructure of the cingulum, corpus callosum and superior longitudinal fasciculus have been associated with sustained (maintenance of focus over time) (Chiang et al., 2015; Klarborg et al., 2013; Santhanam et al., 2011), focused (filtering out distractors) (Ge et al., 2013) and shifting (disengaging and refocusing) (Ursache et al., 2016) attention, as well as behavioral hyperactivity and impulsivity (Chiang et al., 2020; Nagel et al., 2011). The brain regions associated with attention among typically developing children and children with Attention Deficit and Hyperactivity Disorder (ADHD), are regions that are vulnerable to the neurotoxicity of PAE. Altered white matter microstructure in varying brain regions may therefore underpin different dimensions of attention problems in those with PAE. Preliminary evidence identified a link between focused attention and white matter microstructure in the splenium and isthmus of the corpus callosum in children with FASD (Fan et al., 2016), but this is not a universal finding (Wozniak et al., 2009).

Past investigations of white matter microstructure most commonly use the diffusion tensor modelling technique. Diffusion tensor imaging-derived measures such as fractional anisotropy are non-specific to white matter properties (e.g., axon density) and are affected by extra-axonal signal contamination (Jones et al., 2013). Further, they cannot detect complex fiber geometries (e.g., crossing, bending or fanning) of the multiple fiber populations within image voxels. Relating diffusion tensor imaging measures to underlying biology is therefore not ideal (Jones et al., 2013). A more novel technique, Fixel-Based Analysis (FBA), enables the analysis of individual fiber populations within each voxel (fixel), allowing for a finer unit of analysis and more biologically interpretable measures (Dhollander et al. 2021a, b; Raffelt et al. 2015). FBA analyses fiber-specific measures including the microstructural density (fiber density, FD), macrostructural cross-sectional area (fiber cross-section, FC) and the combined fiber density and cross section (FDC) of white matter. FDC reflects the total intra-axonal volume of fibers and their capacity to transmit information (Raffelt et al., 2017). Studies in populations with neurodegenerative and demyelinating disorders or those born preterm demonstrate that greater cross section and density of fibers support cognition (Dhollander et al. 2021a, b). We have recently reported data from the Asking Questions About Alcohol in Pregnancy (AQUA) cohort confirming that low-moderate PAE throughout pregnancy leads to widespread changes in structural brain connectivity (Liang et al., 2024) and reduced FBA-derived cross-sectional area of the cingulum bundle compared with controls (Thompson et al., 2024). However, the implications of these subtle changes in white matter micro- and macro-structure for attention functioning warrant further investigation.

We aimed to apply FBA to examine associations between attention (focus, sustain, shift, behavioral hyperactivity and inattentiveness) and white matter micro- and macro- structure (FD, FC, and FDC) in a cohort of children exposed to different levels of PAE: No exposure; low-moderate PAE only in trimester 1 (T1); low-moderate PAE exposure throughout pregnancy (T1-T3). Based on past literature, including FASD research, it was hypothesized that higher FD, FC and FDC in the cingulum, corpus callosum and superior longitudinal fasciculus would be associated with better attention in control and PAE groups. We secondly aimed to examine whether associations between white matter abnormalities and attention varied across different PAE exposure groups, and hypothesized that the effects of PAE would be dependent on both dose and timing (Guerri, 1998).

## Methods

### Participants

Pregnant women were recruited into the AQUA cohort at their first antenatal appointment from seven hospitals around Melbourne between 2011 and 2012.

#### Inclusion criteria at recruitment

Eligibility criteria included being < 19 weeks pregnant, singleton pregnancy, maternal age ≥16, and English language proficiency sufficient to obtain informed consent and complete questionnaires. Women were required to be attending low-risk public hospital antenatal clinics at the time of recruitment. “Low-risk” status was determined by the attending clinic classification and excluded women with substance dependence, major psychiatric conditions, or pre-existing complex medical conditions requiring specialized obstetric care at baseline. However, women who subsequently developed pregnancy complications (such as preeclampsia) during the course of their pregnancy remained in the cohort. The sample comprised generally well-educated mothers, with 57% having completed a university degree at baseline. Comprehensive data were collected on maternal medical conditions, substance use (including detailed alcohol consumption patterns), diet and supplement intake, as well as demographic and family factors (Muggli et al., 2014).

#### Exclusion criteria at 6–7 year follow-up

For the present analysis conducted at the 6–7 year follow-up, we excluded lifelong alcohol abstainers because our target population was children of mothers who normally drink some alcohol.

At baseline, the AQUA cohort consisted of 1570 children (lifetime abstainers *n* = 112; no PAE *n* = 494; PAE T1-T3 *n* = 461; PAE T1 *n* = 430; and irregular PAE *n* = 73 (Muggli et al., 2016). The cohort was reviewed at ages one (Muggli et al., 2017), two (Halliday et al., 2017), and 6-to-9 years (Muggli et al. 2022a) for craniofacial analysis and/or neuropsychological assessment (*n* = 698).

During the 6–7-year follow-up, 146 children had an MRI, of which 129 children had diffusion imaging data for FBA. Selection was stratified to ensure approximately equivalent numbers with no PAE (controls *n* = 37), PAE T1 (*n* = 47), and PAE T1-T3 (*n* = 45). Approval was granted by the Human Research Ethics Committee at the Royal Children’s Hospital Melbourne (approval #38025) and written parental consent was obtained.

### Procedure

Children completed a neuropsychological assessment at the Murdoch Children’s Research Institute, Melbourne. Assessors were registered or student psychologists blinded to PAE. Children from each subgroup (controls, PAE T1, PAE T1-T3) were invited for MRI at the Royal Children’s Hospital, Melbourne. Mock MRI training was undertaken prior to their MRI to familiarize children with the procedure and MRI was performed on non-sedated children.

### Assessment of PAE

PAE was prospectively measured using questionnaires administered at three timepoints in pregnancy (Muggli et al., 2010, 2014). Information about the type, volume, and frequency of drinking was collected, and converted to grams of absolute alcohol (gAA). The PAE T1 group had 24.1 gAA per week prior to pregnancy recognition, with 0.1 gAA per week on average post pregnancy recognition and included nine with binge-level PAE prior to pregnancy recognition. The PAE T1-T3 group had 39.2 gAA per week on average prior to pregnancy recognition and < 11 gAA per week throughout the remainder of pregnancy and included 13 with binge-level PAE prior to pregnancy recognition. A binge drinking episode was defined as ≥ 50 gAA per occasion (Muggli et al. 2022a, b). Children whose mothers were abstinent from alcohol throughout pregnancy comprised the control group.

### Attention measures and covariates

Attention measures are summarized in Table 1. We evaluated four domains of attention: focus, sustain, shift and behavioral hyperactivity, impulsivity and inattentiveness using validated tools (Table 1).

**Table 1:** Attention measures

Attention domain	Scale/subtest
Focus	Balloon Hunt 5 from the Test of Everyday Attention (TEA-Ch2) (Manly et al., 2016) estimated focused attention. This is a speeded cancellation task. Raw scores, equal to the number of crossed targets divided by the time, were converted to scaled scores (*M* = 10, *SD* = 3) based on the child’s age and sex, with higher scores reflecting superior focused attention.
Sustain	Barking from the TEA-Ch2 (Manly et al., 2016) required children to count tones over 10 trials. Correct responses were converted to scaled scores based on the child’s age and sex (*M* = 10, *SD* = 3), with higher scores reflecting better sustained attention.
Shift	The Contingency Naming Test, trial three measured shifting attention (Taylor et al., 1987). An array of shapes (circle, triangle and square) with smaller internal shapes of differing colors (pink, green and blue) were presented and were named according to a rule. Efficiency scores were used in analyses: efficiency= ($\:\frac{\frac{1}{time\left(seconds\right)}}{\sqrt{number\:oferrors+1}}$) x 100 (Anderson et al., 2000).
Behavioral hyperactivity, impulsivity and inattentiveness	The Attention Deficit Hyperactivity Disorder Rating Scale (ADHD-RS-5) (DuPaul et al., 2016) is an 18-item questionnaire. Parents rated their child’s home behavior on a 4-point scale (0, never/rarely to 3, very often). Scores were summed and converted to percentile ranks based on the child’s age and sex, with higher scores indicative of behavioral attention problems.

Social risk, child age and sex, intracranial volume and binge PAE were included as covariates (Ghazi Sherbaf et al., 2019; Suades-Gonzalez et al., 2017; Ursache et al., 2016). Social risk was estimated by the Social Risk Index which measured: family structure, education of the primary caregiver, occupation and employment of the primary income earner, and maternal language and age at the child’s birth (Roberts et al., 2008). Raw scores were dichotomized around the median (lower social risk 0–1; higher social risk ≥2–12).

### MRI acquisition

MRI data were acquired on a Siemens Magnetom Prisma 3 T scanner. Diffusion-weighted MRI data were acquired with 60 gradient directions, *b*-value = 2800 s/mm^2^, 10 *b* = 0 s/mm^2^ images, 2 mm isotropic voxels, multi-band acceleration factor = 2, repetition time = 3500 ms, echo time = 67 ms. An additional pair of *b* = 0 s/mm^2^ images with opposite phase encodings were acquired to correct for echo planar imaging-induced geometric distortions in the diffusion MRI data. *T*_*1*_-weighted images were also acquired (3D magnetization-prepared gradient-echo (MP-RAGE) sequences with prospective motion correction, echo times = 2.14, 3.94, 5.77 and 7.5ms, repetition time = 2550 ms, Field of View = 256 × 256 mm^2^, matrix = 288 × 288, 208 sagittal slices, slice thickness = 0.9 mm, interpolated voxel size = 0.9 × 0.4 × 0.4 mm^3^) and were used to generate a measure of intracranial volume for statistical analyses, using the standard FreeSurfer version 7.1.1 recon-all pipeline (Fischl, 2012).

### Diffusion MRI processing

Diffusion MRI data were processed predominantly using MRtrix3 version 3.0.1 (Tournier et al., 2012), MRtrix3Tissue (https://3Tissue.github.io, a fork of MRtrix3; version 5.2.8), and Functional MRI of the Brain Software Library (FSL 6.0.3) (Jenkinson et al., 2012), following all steps in the typical FBA pipeline (Dhollander et al. 2021a, b). Pre-processing included correction for Gibbs-ringing artefact (Kellner et al., 2016), participant movement, eddy current-induced distortions, susceptibility-induced distortions and susceptibility-by-movement interactions (Andersson et al., 2016, 2017, 2018; Andersson & Sotiropoulos, 2016). Image quality control was performed by visual inspection and automatically using the FSL QUAD and SQUAD tools (Bastiani et al., 2019).

The following steps were performed: estimation and averaging of 3-tissue response functions (Dhollander et al., 2019), upsampling to 1.5 mm isotropic voxels, Single-Shell 3-Tissue Constrained Spherical Deconvolution (SS3T-CSD) (Dhollander & Connelly, 2016), and bias field correction and global intensity normalization (Thijs Dhollander et al. 2021a, b). These steps resulted in the white matter fiber orientation distribution, grey matter, and cerebrospinal fluid compartment images for each participant. A study-specific white matter fiber orientation distribution template was created by iterative registration and averaging of a random selection of 30 participants’ fiber orientation distribution images from each group, following which all participants’ images were registered and warped to the template (Raffelt et al., 2011). For each participant in template space, fixels were segmented and reorientated, and correspondence with common template fixels was established. Finally, the fixel-wise FD, FC (in log form) and FDC metrics were computed (Raffelt et al., 2017).

### Statistical analyses

Demographic and attention variables were analyzed using two-sample *t*-tests for continuous variables and chi squared tests for categorical variables in Stata Statistical Software Release 16 (StataCorp., 2019).

General Linear Models assessed associations between attention and FD, FC and FDC per subgroup (Control, PAE T1, PAE T1-T3) at the fixel-wise level. Analyses were adjusted for age at MRI, sex, social risk, and intracranial volume, which was log-transformed and only included in FC and FDC models (Smith et al., 2019). Connectivity based smoothing and statistical inference were performed using connectivity-based fixel enhancement (Raffelt et al., 2015). Non-parametric permutation testing with 5000 permutations were used to generate family-wise error rate (FWE) corrected *p*-values per fixel.

Interaction analyses investigated whether the association of FD, FC and FDC metrics with attention differed *between* groups (controls versus PAE T1 versus PAE T1-T3), using General Linear Models adjusted for age at MRI, sex, social risk, and intracranial volume for FC and FDC models. Connectivity-based fixel enhancement, and non-parametric permutation testing were performed. For interpretation of significant group-by-attention interactions, FD, FC and FDC were extracted from fiber pathways (Wasserthal et al., 2018) and associated with attention in each group.

Finally, we conducted sensitivity analyses that adjusted for binge PAE. These analyses followed the same approach as above.

## Results

Of the 146 children who had an MRI, 14 were excluded due to incomplete acquisition of diffusion MRI and three were excluded due to movement artefacts. The final sample comprised 129 children (66 female, 63 male) of mean age 7.2 (SD 0.2) years (controls, *n* = 37; PAE T1 *n* = 47; PAE T1-T3 *n* = 45). Table 2 displays their demographic characteristics. Mothers were predominantly aged 30 years or older at birth (76%), highly educated (69% tertiary), and of normal or underweight BMI pre-pregnancy (60%). Controls had a higher social risk background than the PAE T1 group. Prenatal alcohol exposure patterns varied across trimesters. In the PAE T1 group, exposure to alcohol dropped to almost zero once mothers became aware of their pregnancy. Prior to pregnancy recognition, mean weekly exposure was 24.1 g of absolute alcohol (gAA) (SD = 73.7) with a mean maximum exposure of 32.1 gAA per occasion (SD = 35.3). One standard drink in Australia equates to 10 gAA; these exposure levels translate to approximately 2.4 standard drinks per week with a maximum of 3.2 standard drinks per occasion. To contextualise, 3.2 standard drinks is equivalent to approximately 2.2 glasses (150 ml) of 12% wine. Almost one in five children (18%) in the PAE T1 group were exposed to at least one binge-level episode (≥ 5 standard drinks per occasion) during this period.

**Table 2:** Participant characteristics

	Controls	PAE T1	PAE T1-T3
*N (%)*	37 (28.7)	47 (36.4)	45 (34.9)
Maternal characteristics			
Maternal age at birth N (%)			
< 25 years	2 (5.4)	1 (2.1)	0 (0)
25–29 years	7 (18.9	13 (27.7)	8 (17.8)
30–34 years	14 (37.8)	14 (29.8)	20 (44.4)
≥ 35 years	14 (37.8)	19 (40.4)	17 (37.8)
Maternal education N (%)			
High school	2 (5.4)	6 (12.8)	1 (2.2)
Trade/diploma	14 (37.8)	8 (17.0)	9 (20.0)
University	21 (56.8)	33 (70.2)	35 (77.8)
Pre-pregnancy BMI N (%)			
Normal/underweight (< 25)	19 (54.3)	28 (62.2)	27 (61.4)
Overweight (25–29.9.9)	6 (17.1)	8 (17.8)	11 (25.0)
Obese (≥ 30)	10 (28.6)	9 (20.0)	6 (13.6
Higher social risk background N (%)	17 (46.0)^a^	11 (23.4)^a^	15 (33.3)
Prenatal Alcohol exposure			
Trimester 1, pre-awareness			
Mean gAA/week (SD)		24.1 (73.7)	39.2 (51.5)
Mean gAA/Occasion (SD)		32.1 (35.3)	36.1 (37.2)
Binge episode N (%)		9 (19.1)	13 (28.9)
Trimester 1, post-awareness			
Mean gAA/week (SD)		0.1 (0.5)	7.4 (20.8)
Mean gAA/Occasion (SD)		0.9 (2.7)	7.2 (9.1)
Binge episode N (%)			Nil
Trimester 2			
Mean gAA/week (SD)			10.0 (20.2)
Mean gAA/Occasion (SD)			9.7 (7.1)
Binge episode N (%)			Nil
Trimester 3			
Mean gAA/week (SD)			6.2 (10.5)
Mean gAA/Occasion (SD)			8.4 (6.7)
Binge episode N (%)			Nil
Child characteristics			
Child age (years) at MRI, *M* (*SD*)	7.1 (0.2)	7.2 (0.2)	7.2 (0.2)
Child sex (female), *N (%)*	18 (48.6)	29 (61.7)	19 (42.2)
Total intracranial volume (cm^3^), *M* (*SD*)^b^	1503 (106)	1539 (143)	1598 (122)
Total brain tissue volume (cm^3^), *M* (*SD*)^b^	1181 (98)	1199 (108)	1243 (99)
Total cortical volume (cm^3^), *M* (*SD*)^b^	586 (52)	593 (54)	618 (47)
Total white matter volume (cm^3^), *M* (*SD*)^b^	399 (42)	405 (47)	420 (45)
Focused attention standard score, *M* (*SD*)	9.2 (3.0)	8.5 (2.9)	9.6 (2.8)
Shifting attention efficiency score, *M* (*SD*)^c^	1.2 (0.43)	1.13 (0.37)	1.20 (0.44)
Sustained attention standard score, *M* (*SD*)^c^	9.7 (3.1)	9.4 (2.8)	10.2 (3.0)
Hyperactive subscale percentile rank, *M* (*SD*)	46.8 (35.1)	39.2 (26.4)	44.5 (30.8)
Inattentive subscale percentile rank, *M* (*SD*)	45.2 (31.8)	41.7 (26.8)	50.6 (28.2)

*GAA* grams of absolute alcohol; *SD* standard deviation; *M* mean

^a^*p*<0.05 for difference between controls and PAE T1 group, ^b^Volumes were derived from FreeSurfer software, as previously described(Thompson et al., 2024), ^c^sample size for controls *n* = 36 for the analysis due to missing data

The PAE T1-T3 group showed continued exposure throughout gestation. Prior to pregnancy recognition, mean weekly exposure was 39.2 gAA (SD = 51.5) with a mean maximum exposure of 36.1 gAA per occasion (SD = 37.2), equivalent to approximately 2.5 glasses of wine per occasion. Approximately 3 in 10 children (29%) were exposed to at least one binge-level episode during this period. Following pregnancy recognition, exposure levels dropped substantially, with mean weekly levels ranging from 6.2 to 10.0 gAA and mean maximum levels per occasion ranging from 6.2 to 9.7 gAA across trimesters two and three. Maximum exposure did not reach 50 gAA per occasion in trimesters two and three (i.e., no binge-level exposure). There were no differences in age, sex or attention between groups. Controls had a higher social risk background than the PAE T1 group.

### Associations between attention and white matter within each group

**Fig. 1:**
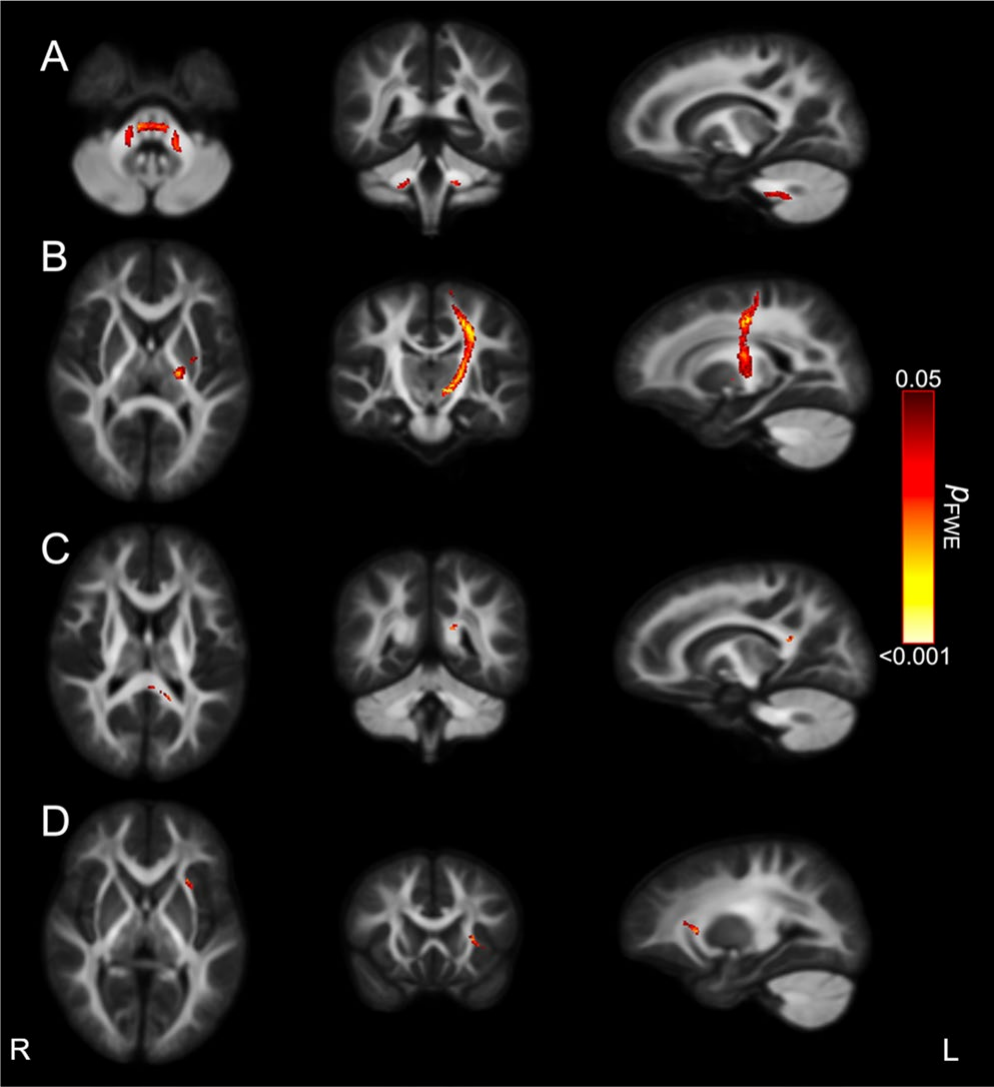
Associations between attention domains and fixel-based metrics. Figures were generated by highlighting streamline segments from the whole-brain tractogram that passed through fixels that were significantly associated with attention performance (*p*_*FWE*_<0.05). Streamlines were colored by *p*_*FWE*_ value. Figures illustrate the relationship between (**A**) fewer hyperactive symptoms with higher FC for Control group; (**B**) higher focused attention with higher FC for the Control group; (**C**) higher inattentiveness with higher FD for the PAE T1 group; (**D**) higher inattentiveness with lower FC for the PAE T1 group. All significant results were obtained from models that adjusted for intracranial volume (for FC and FDC), age, sex and social risk

In controls, greater FC in the cerebellar white matter and the corticospinal tract were associated with less hyperactivity and better focused attention, respectively (Fig. 1A and B). In the PAE T1 group, greater FD in the splenium of the corpus callosum and lower FC in the external capsule were associated with higher inattentiveness (Fig. 1C and D).

### Differences in white matter-attention associations between groups

**Fig. 2:**
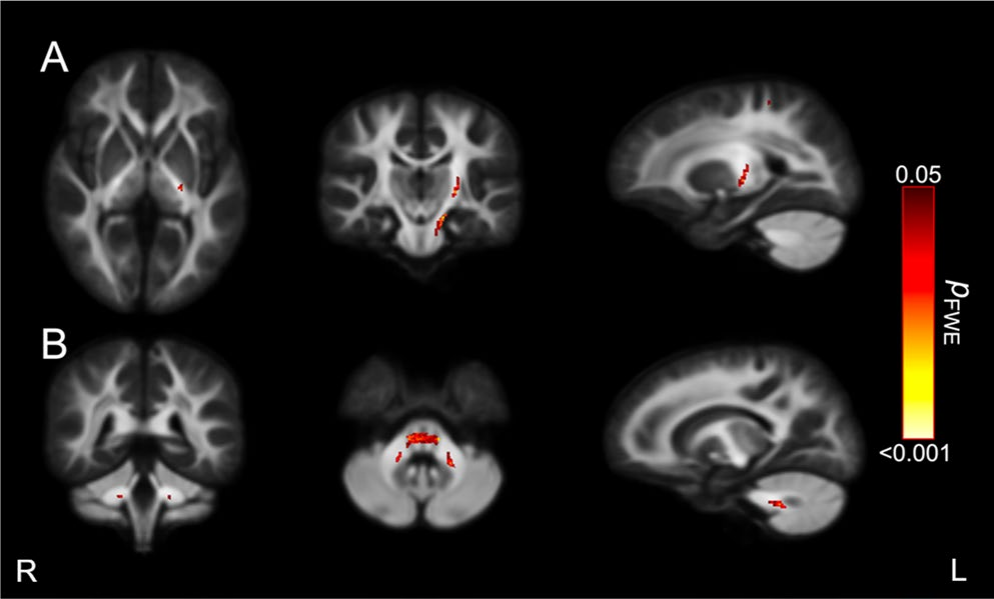
Group-by-attention interactions in fixel-based metrics. Figures were generated by highlighting streamline segments from the whole brain tractogram that passed through fixels that exhibited a significant group-by-attention interaction (*p*_*FWE*_<0.05). Streamlines were colored by *p*_*FWE*_ value. Figures depict significant group-by-attention interaction effects for, (**A**) FDC and focused attention for the Control versus the PAE T1 group; and (**B**) for FC and hyperactive attention for the Control versus the PAE T1-T3 group. All significant results were obtained from models that adjusted for intracranial volume (for FC and FDC), age, sex and social risk

**Fig. 3:**
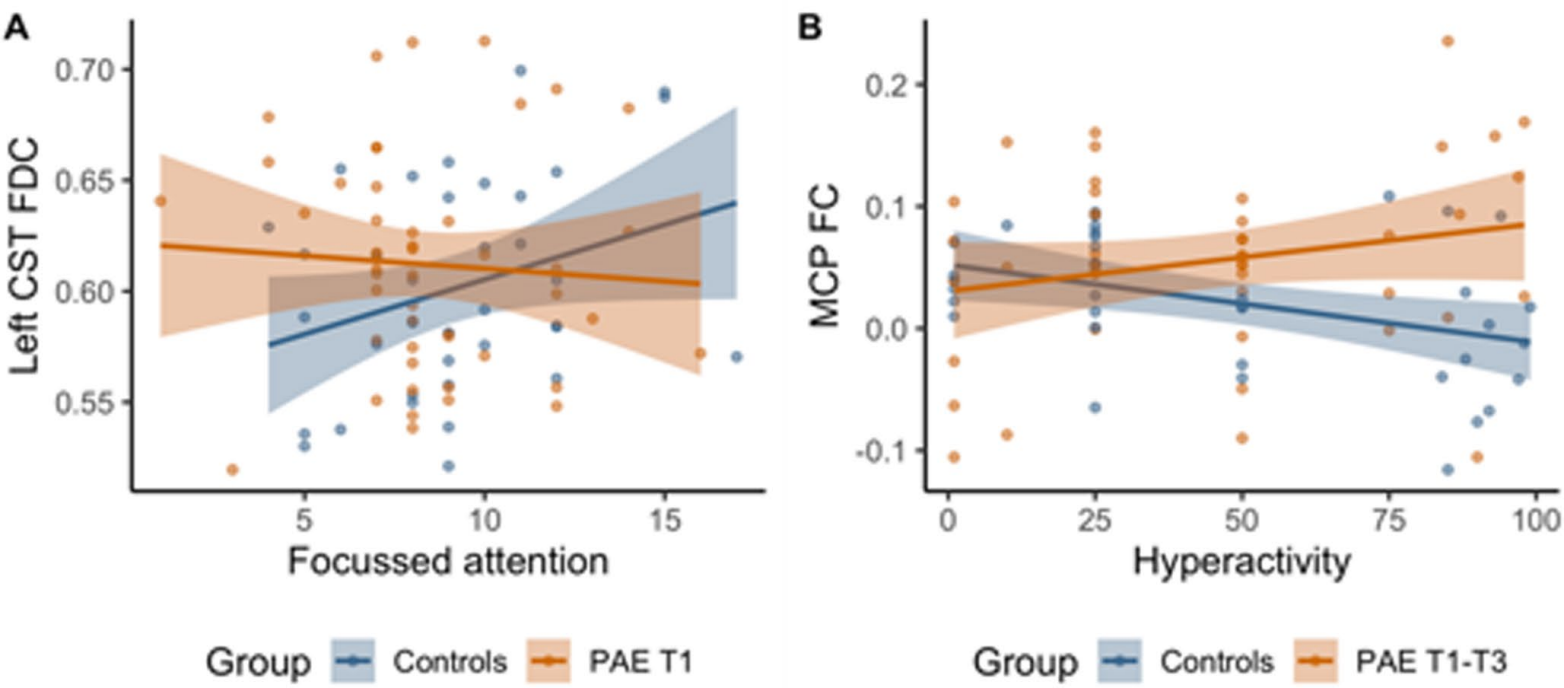
Scatterplot of significant interaction effects. Associations between (**A**) FDC of the left corticospinal tract (CST) and focused attention separately for the control and PAE T1 groups; (**B**) FC of the middle cerebellar peduncle (MCP) and hyperactivity separately for the control and PAE T1-T3 groups. Plots demonstrate that for the control group (in blue), higher FBA metrics (FDC and FC) were strongly associated with greater focused attention and less hyperactivity (*p* = 0.004 and *p* = 0.003). Alternatively, in the PAE groups (in orange) weaker opposite direction associations were seen (*p* = 0.7 for focused attention and *p* = 0.01 for hyperactivity)

There was a significant group interaction effect (controls vs. PAE T1 group) for the association between FDC in the left corticospinal tract and focused attention (Fig. 2A). There was also a significant group interaction effect (controls vs. PAE T1-T3 group) for the association between FC in the cerebellar white matter and hyperactivity (Fig. 2B). Both significant interaction effects highlighted the strong relationships between white matter metrics and attention metrics in the control group, whereas the relationships between white matter metrics and attention metrics were weaker in the PAE groups (Fig. 3).

### Sensitivity analyses adjusted for binge PAE

After adjustment for binge PAE, the association between greater FD in the splenium and higher inattentiveness in the PAE T1 group remained (Supplementary Fig. 1), but otherwise there were no significant associations between attention and white matter within the PAE groups. Interactions were consistent with the above findings but spanned a larger spatial extent (Supplementary Fig. 2).

## Discussion

To understand the biological and clinical implications of low-moderate alcohol consumption patterns during pregnancy, we investigated the relationship between whole-brain white matter micro- and macro-structure and attention in children. There was evidence that specific pathways in white matter were associated with attention. In controls, greater white matter FC in the cerebellum was associated with less hyperactivity and in the corticospinal tract with better focused attention. For the PAE T1 group, greater FD in the splenium of the corpus callosum was associated with greater inattentiveness, while reduced FC in the external capsule was associated with higher inattentiveness. However, the latter finding did not persist after controlling for binge PAE. There was some evidence that associations differed between PAE and control groups. Relationships were stronger in the control than PAE T1group for associations between higher FDC in the left corticospinal tract and focused attention. Relationships were also stronger for controls than the PAE T1-T3 group for associations between higher FC in the cerebellar white matter with less hyperactivity. These interactions persisted after controlling for binge PAE.

Associations between the structure of the cerebellum and hyperactivity suggest that a smaller cross-sectional area of these fibers underlies a reduced capacity for typically developing children to regulate overactivity. This is consistent with past literature on the role of the cerebellum in movement (Ramnani, 2006), behavioral regulation (Baillieux et al., 2008), executive function (Salman & Tsai, 2016) and attention (Ge et al., 2013).

The association between lower FDC in the corticospinal tract and focused attention in controls suggests a slower capacity of corticospinal tract fibers to relay information, leading to poorer performance. Although the corticospinal tract is not typically implicated in attentional processes, its involvement in this study may reflect the fine motor and response time aspects of the task given evidence of this somatosensory tract’s role in bimanual coordination (Fuelscher et al., 2021) and sensorimotor speed (Karahan et al., 2019). Lateralization to the left is consistent with previous findings that manual dexterity is associated with FC on the contralateral side to the predominant hand (Fuelscher et al., 2021). Biologically, lower FDC reflects a slower capacity of corticospinal tract fibers to relay information, leading to poorer performance.

Neuropathology in the posterior regions of the corpus callosum, particularly the splenium, has been identified in individuals with FASD and associated with cognitive deficits including in verbal learning and executive dysfunction (Ghazi Sherbaf et al., 2019). Thinning, hypoplasia and volume loss of the splenium in children with FASD (Spadoni et al., 2007), as well as dose-response associations between diffusion tensor imaging metrics and FASD severity (Fryer et al., 2009; Li et al., 2009), have led to suggestions that this region is particularly vulnerable to PAE (Ghazi Sherbaf et al., 2019). Our results extend findings by implicating this structure in attention difficulties for children with PAE in T1 only. Unexpectedly, higher FD was associated with increased inattentiveness. While counterintuitive, this may align with previous reports linking executive dysfunction to corpus callosum thickening in FASD populations (Bookstein et al., 2002). The biological underpinnings of this association remain unclear, as several mechanisms could contribute to the observed effect such as axonal atrophy, increased intra-cellular volume, or age-related differences in white matter maturation. Accordingly, these findings should be interpreted with caution (Dhollander et al. 2021a, b).

The cingulum and superior longitudinal fasciculus have previously been associated with attention (Chiang et al., 2015; Donald et al., 2015), which was contradictory to our current findings and hypotheses. Associations may be less widespread because FBA is more biologically specific and robust to fiber geometry than other techniques previously used (Raffelt et al., 2015). The superior longitudinal fasciculus has many fiber populations of differing orientations which likely confounded previous relationships (Schilling et al., 2017). FBA investigations have similarly found constricted associations, even in populations with more extensive white matter injury (Bauer et al., 2020; Wallace et al., 2020).

Other than the differences in FDC in the left corticospinal tract and FC in the cerebellar white matter, there was limited evidence that associations differed based on PAE timing. Moreover, data across groups showed substantial overlap when examining interaction effects, suggesting minimal divergence in white matter measures. Large variance in cognition is needed to detect differences related to the timing of PAE (Lebel et al., 2011). Limited variance in our sample may suggest a weak effect of low-moderate PAE patterns on the white matter that subserves attention. Additionally, PAE groups were classified based on exposure timing (T1 only versus throughout T1-T3). Future research would benefit from larger cohorts with the capacity to utilize more objective statistical classification, such as group-based trajectory modelling (Nagin & Odgers, 2010).

Technical limitations of our study are important to consider when interpreting the findings. Notably, we observed no group differences in attention scores, and the range of scores across all attention measures was narrow. This restricted variability may have limited the sensitivity of these measures to detect subtle effects of PAE, particularly in sustained attention where group performance was above average. Furthermore, the sustained attention task was auditory in nature, which is reportedly less affected by PAE (Ware et al., 2021). Future research should therefore investigate visual sustained attention. Several neurobiological and epigenetic factors that may have influenced the results were not assessed such as maternal substance use and nutritional characteristics. Additionally, our exposure measures did not account for individual differences in maternal alcohol metabolism (influenced by factors such as age, weight, and genetic variations in metabolizing enzymes), which may have introduced measurement error and attenuated the strength of observed associations. Maternal education levels were higher in the PAE groups compared with controls, consistent with the larger AQUA cohort demographics. As higher maternal education is typically associated with better child neurodevelopmental outcomes, this pattern would be expected to bias results toward the null, making our detected associations between PAE and white matter microstructure conservative estimates of true effects. Nonetheless, these limitations suggest that our findings should be considered preliminary and interpreted with caution.

## Conclusion

This study provides insights into the neurobehavioral sequelae of low-moderate PAE patterns by delineating white matter correlates of attention skills in children with and without PAE. In those without PAE, greater cross-sectional area of cerebellum fibers was associated with less hyperactivity and in the corticospinal tract with better focused attention. In those with PAE in T1 only, higher density of the splenium fibers was associated with more inattentiveness, while reduced cross-sectional area of external capsule fibers was associated with more inattentiveness. There was evidence of subtle group differences in the associations between white matter structure and attention, where relationships were stronger in control than PAE groups. Further research is required to determine the reproducibility of these preliminary findings.

## Supplementary Information

Below is the link to the electronic supplementary material.

ESM 1 (DOCX 546 KB)

## Data Availability

As the study is ongoing, data will not be publicly available.

## Abbreviations

ADHD: Attention Deficit and Hyperactivity Disorder
AQUA: Asking Questions About Alcohol in Pregnancy cohort
FASD: Fetal Alcohol Spectrum Disorder
FBA: Fixel–Based Analysis
FC: Fiber Cross Section
FD: Fiber Density
FDC: Fiber Density and Cross Section
FSL: Functional MRI of the Brain Software Library
FWE: family–wise error rate
PAE: Prenatal Alcohol Exposure
T1: Trimester 1 only
T1-T3: Trimester 1 through to Trimester 3 (throughout pregnancy)

## Code availability

As the study is ongoing, code will not be publicly available.
