# Supplementary material for: Associations between white matter micro- and macro-structure and attention in 6-7-year-old children with low to moderate prenatal alcohol exposure
Source: Brain Imaging Behav. 2026 Feb 25;20(2):22. doi: 10.1007/s11682-026-01110-4 (PMC12935816; doi:10.1007/s11682-026-01110-4)
Supplement: Supplementary file 1 — (DOCX 546 KB) [file 11682_2026_1110_MOESM1_ESM.docx]

**Supplementary Material**


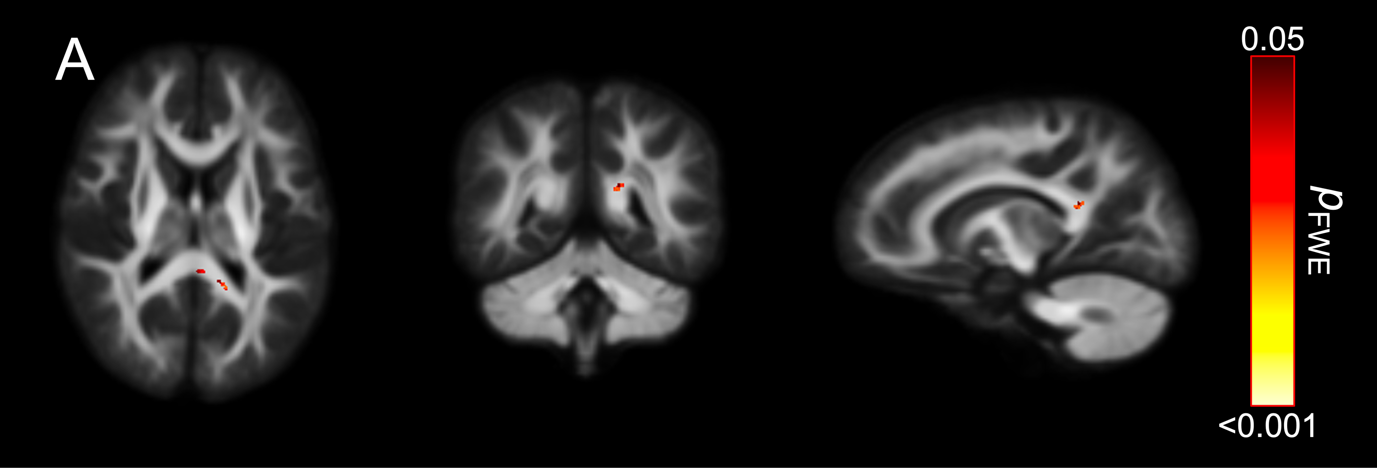


**Figure S1. Associations between attention domains and fixel-based metrics adjusted for binge PAE**

Figures were generated by highlighting streamline segments from the whole-brain tractogram that passed through fixels that were significantly associated with attention performance (*p_FWE_*<.05). Streamlines were coloured by *p_FWE_* value. Figures illustrate the relationships between A) higher inattentiveness with higher FD for the PAE T1 group. All significant results were obtained from models that adjusted for age, sex, social risk and binge PAE.

**
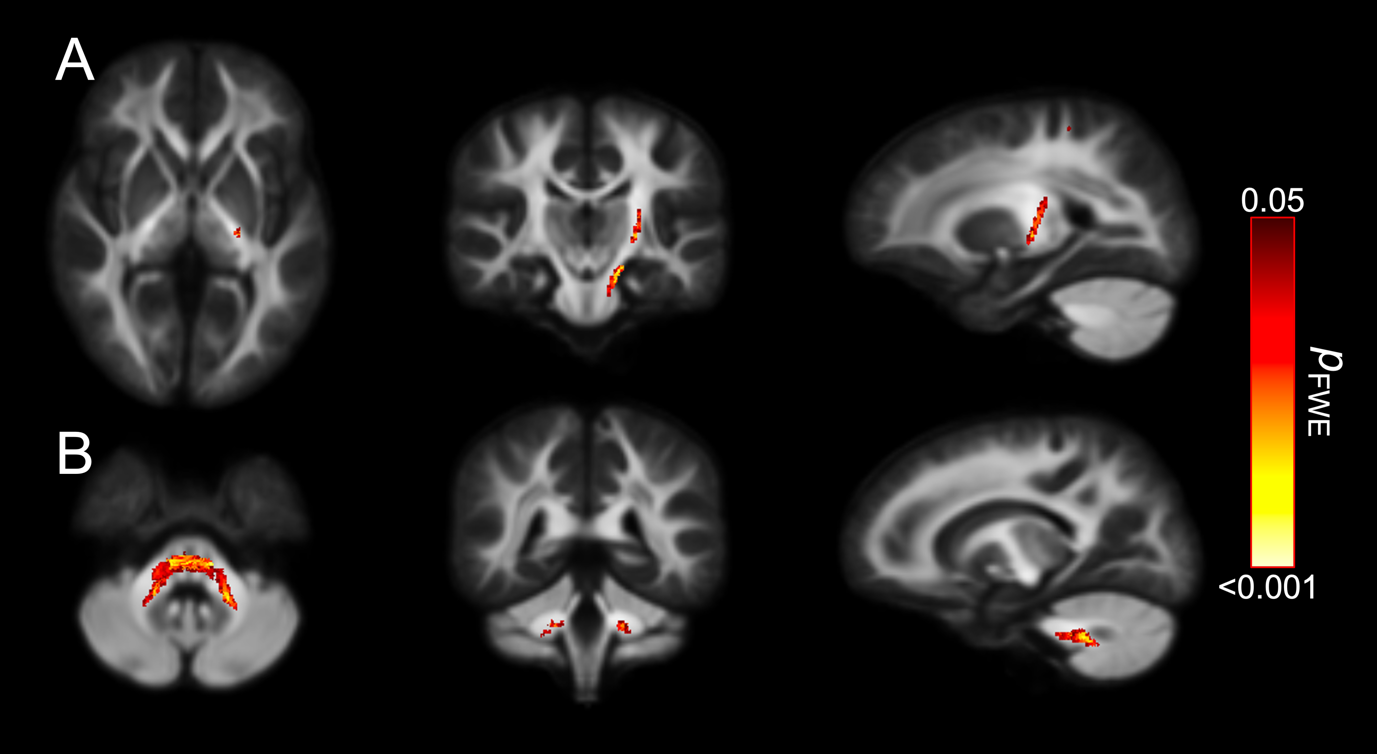
**

**Figure S2. Group-by-attention interactions in fixel based metrics adjusted for binge PAE**

Figures were generated by highlighting streamline segments from the whole brain tractogram that passed through fixels that exhibited a significant group-by-attention interaction (*p_FWE_*<.05). Streamlines were coloured by *p_FWE_* value. Figures depict significant group-by-attention interaction effects for, A) FDC and focused attention for the Control versus the PAE T1 group; and B) for FC and hyperactive attention for the Control versus the PAE T1-T3 group. All significant results were obtained from models that adjusted for intracranial volume (for FC and FDC), age, sex, social risk and binge PAE.
